# Improving Current Glycated Hemoglobin Prediction in Adults: Use of Machine Learning Algorithms With Electronic Health Records

**DOI:** 10.2196/25237

**Published:** 2021-05-24

**Authors:** Zakhriya Alhassan, Matthew Watson, David Budgen, Riyad Alshammari, Ali Alessa, Noura Al Moubayed

**Affiliations:** 1 Department of Computer Science Durham University Durham United Kingdom; 2 College of Computer Science and Engineering University of Jeddah Jeddah Saudi Arabia; 3 National Center for Artificial Intelligence Saudi Data and Artificial Intelligence Authority Riyadh Saudi Arabia; 4 Department of Information Technology Programs Institute of Public Administration Riyadh Saudi Arabia

**Keywords:** glycated hemoglobin HbA_1c_, prediction, machine learning, deep learning, neural network, multilayer perceptron, electronic health records, time series data, longitudinal data, diabetes

## Abstract

**Background:**

Predicting the risk of glycated hemoglobin (HbA_1c_) elevation can help identify patients with the potential for developing serious chronic health problems, such as diabetes. Early preventive interventions based upon advanced predictive models using electronic health records data for identifying such patients can ultimately help provide better health outcomes.

**Objective:**

Our study investigated the performance of predictive models to forecast HbA_1c_ elevation levels by employing several machine learning models. We also examined the use of patient electronic health record longitudinal data in the performance of the predictive models. Explainable methods were employed to interpret the decisions made by the black box models.

**Methods:**

This study employed multiple logistic regression, random forest, support vector machine, and logistic regression models, as well as a deep learning model (multilayer perceptron) to classify patients with normal (<5.7%) and elevated (≥5.7%) levels of HbA_1c_. We also integrated current visit data with historical (longitudinal) data from previous visits. Explainable machine learning methods were used to interrogate the models and provide an understanding of the reasons behind the decisions made by the models. All models were trained and tested using a large data set from Saudi Arabia with 18,844 unique patient records.

**Results:**

The machine learning models achieved promising results for predicting current HbA_1c_ elevation risk. When coupled with longitudinal data, the machine learning models outperformed the multiple logistic regression model used in the comparative study. The multilayer perceptron model achieved an accuracy of 83.22% for the area under receiver operating characteristic curve when used with historical data. All models showed a close level of agreement on the contribution of random blood sugar and age variables with and without longitudinal data.

**Conclusions:**

This study shows that machine learning models can provide promising results for the task of predicting current HbA_1c_ levels (≥5.7% or less). Using patients’ longitudinal data improved the performance and affected the relative importance for the predictors used. The models showed results that are consistent with comparable studies.

## Introduction

### Background

The level of glycated hemoglobin (HbA_1c_) is used to measure the average glucose concentration in red blood cells [1,2]. Unlike other glucose blood tests, such as random blood sugar (RBS) and fasting blood sugar (FBS), HbA_1c_ provides a long-term measure of a patient’s blood glucose levels [3]. The HbA_1c_ test can therefore provide physicians with a reliable means of monitoring a patient’s hyperglycemia without requiring the patient to undertake overnight fasting prior to being tested.

A concentration of 6.5% for the HbA_1c_ in patient blood is considered as the cutoff point for the diagnosis of diabetes [4]. However, patients with a concentration of less than 6.5% are not completely excluded from a diabetes diagnosis, as the range of elevation levels (5.7%≤ HbA_1c_ <6.5%) can indicate the future onset of diabetes. Therefore, HbA_1c_ can act as an early predictor for the potential development of type-2 diabetes mellitus (T2DM) [2]. Ackermann et al [3] suggested using the HbA_1c_ test as a measure for identifying those adults who are at a greater risk of developing T2DM in the future.

Research has shown that reducing HbA_1c_ levels can significantly reduce the possibility of developing serious complications. Hence, close monitoring of HbA_1c_ levels is recommended for all diabetic patients and those with the potential for developing diabetes [5]. It is also suggested that diabetic and nondiabetic patients with raised HbA_1c_ levels should be clinically checked and monitored as a preventive intervention to avoid developing T2DM [6].

Currently, the clinical data collected from patient visits consists of a set of readings for vital signs and lab tests, diagnoses, physicians’ notes, and treatments that are stored in electronic health records (EHRs). These are collected on an irregular basis, according to clinical needs, and stored with an associated time stamp.

In recent years, machine learning models have shown powerful capabilities for analyzing and understanding complex data across a wide variety of applications. Our research question for this study was as follows: “Can HbA_1c_ prediction be improved by using machine learning with longitudinal data that are normally available in EHR systems?”

This paper reports an investigation into the performance of machine learning models to predict current HbA_1c_ levels as a binary classification problem using EHR data. Nondiabetic patients with an HbA_1c_ level of 5.7% or more are considered to have an elevated HbA_1c_, while those with levels lower than this are considered normal. The models combine current visit data with extra features (independent variables) extracted from previous visits by patients. We used explainable methods to rank the features in order of their importance to the decision made by each of the models. To the best of our knowledge, this study is the first to employ machine learning models that use longitudinal data from EHR systems for the purpose of HbA_1c_ elevation risk prediction. This study is also the first to use explainable machine learning techniques to explain the classification decisions made by black box models, support vector machine (SVM), and multilayer perceptron (MLP), in predicting HbA_1c_ elevation risk (≥5.7%), in order to better understand the behavior of the model.

### Related Work

EHR data have been intensively investigated for a variety of medical decision support tasks [7]. These tasks include the analysis of complex patterns and prediction of major medical events (for example, diagnostic imaging and gene interactions) [8,9]. Several studies have demonstrated the successful employment of EHR data with prediction models [10]. For instance, machine learning has been intensively used with EHR data in diagnosing diabetes and discovering its related patterns [11-15]. However, we are not aware of any studies that have explored machine learning models for the prediction of current elevated HbA_1c_ levels using EHR data from a nondiabetic population or the impact of patient longitudinal data on the effectiveness of such predictive machine learning models.

Several studies have investigated the association between HbA_1c_ levels and clinical variables using statistical models [16,17]. A study by Rose et al [18] discussed the correlation between RBS and HbA_1c_ levels. Stanley et al [19] used a linear regression model for imputation of missing HbA_1c_ data. Their model calculates HbA_1c_ levels for patient records with missing HbA_1c_ values as continuous and categorical values and uses 4 predictors extracted from an EHR system—RBS, FBS, age, and gender—as predictors to calculate the level of HbA_1c_ for a diabetic population. Simone et al [20] used linear regression models to predict HbA_1c_ levels after 6 years for nondiabetic patients using different populations.

A study by Wells et al [21] in 2018 was the first to focus on predicting current HbA_1c_ elevation levels for nondiabetic patients through use of an EHR data set. Multiple logistic regression (MLR) was employed to calculate the probability of a patient having an elevated HbA_1c_ level (≥5.7%). The data set was extracted from an EHR system used in the United States. The authors used 8 independent variables fitted to the model using restricted cubic splines with 3 knots to formulate the final equation. The performance of the MLR model was compared to that of the models used by Baan et al [22] and Griffin et al [23]. However, the models by Baan and Griffin aimed at predicting the onset of patients’ diabetes rather than predicting HbA_1c_ levels for nondiabetic patients. In addition, the experimental data set used by Wells et al to train and test their model was imbalanced with 74% of the samples having normal HbA_1c_ levels (5.7%) and only 26% of the samples having elevated HbA_1c_ levels (≥5.7%).

We performed a differentiated replication of the study by Wells et al [21] using the more balanced King Abdullah International Medical Research Center (KAIMRC) data set [24]. Although the significant variables identified in our replication were in general agreement with those of the original study, there were some differences in the ranking of importance for these, suggesting that such models do need to be “tuned” to the characteristics of different populations.

## Methods

### Study Design

To study the impact of using advanced predictive models with EHR data to predict current HbA_1c_ levels, we employed the MLR, random forest (RF), SVM, and logistic regression (LR) models, as well as a deep learning model, MLP [25]. The problem was formulated into a binary classification problem whereby the target variable, HbA_1c_ level, was encoded as 1 when the level of HbA_1c_ was 5.7% or more and with 0 otherwise. The results obtained from using these models were compared to those obtained from employing the model used by Wells et al with the KAIMRC data set (detailed in the Data Set subsection).

The performance of the models was investigated using current visit data only and with additional longitudinal data from current and previous visits. The performance of each model was evaluated using measures commonly employed in clinical applications. For the SVM and MLP models, the relative importance of the features was also calculated using explainable machine learning techniques.

### Explainable Methods for Black Box Models

Using black box machine learning models in health care can have adverse effects on the trust and confidence placed in their outcomes; the risk of misclassification is potentially too high for clinicians to confidently use black box models for high risk health care decisions, and not being able to interpret a model’s decision exacerbates this problem [26]. Explainable methods for machine learning models allow interpretable outcomes that can expose the reasons behind the decision made by the model [27]. This transparency provides both health professionals and patients with the confidence and trust in the outcome of the models. The widely used Shapley Additive Explanations (SHAP) values [28] and local interpretable model-agnostic explanations (LIME) score [29] techniques have therefore been used to provide a degree of transparency to our deep learning model.

SHAP values are derived from Shapley values used in game theory and provide a method of calculating the contribution of each feature (variable) to the final prediction via the GradientSHAP approximation. This is achieved for each feature by comparing the prediction the model makes when the feature is present with the prediction obtained when the feature takes some baseline value [28]. Consequently, the SHAP values for a given input “explain” how each feature affects the output of the model when compared to the baseline (or “default”) output of the model. We used SHAP values to interpret our black box models, so they could be efficiently calculated, and their use enabled a global view of the model to be constructed through the computation of SHAP values from across the whole data set.

SHAP values were computed using the feature’s mean marginal contribution across different coalitions of all features. SHAP values themselves are computationally intensive to compute, and so approximation methods are commonly used when calculating the values.

To ensure that the SHAP values we calculated were not too greatly affected by the approximation method used, we also computed the LIME [29] scores for the models across the entire data set. LIME tries to estimate locally faithful linear explanations (ie, explanations that correspond to how the model behaves around the instance being explained) for any classifier. LIME achieves this by creating local linear classifiers that approximate the behavior of the original model in the vicinity of the data being explained. As linear models are inherently interpretable through their parameters, they can be used to generate explanations of the original model. Both SHAP and LIME have the advantage that they are model-agnostic techniques, and so we were able to apply both methods to both of our black box classification models (SVM and MLP).

### Data Set

The data used in this study were taken from the KAIMRC data set. The data were collected from King Abdulaziz Medical City located in the central and western regions of Saudi Arabia, an area which has been ranked second in the Middle East and seventeenth in world in diabetes prevalence by the World Health Organization (WHO) [30]. According to the International Diabetes Federation, the diabetes prevalence rate in Saudi Arabia is 18.3%. Therefore, the availability of the data from this population provides considerable opportunities for research into the early prediction of diabetes.

The data set contains a full history of patient details, vital signs, and lab test readings for each patient visit for the period from 2016 to the end of 2018. As the aim of this study was to identify nondiabetic patients that are at a high risk of HbA_1c_ elevation, all patients previously diagnosed with hyperglycemia were excluded from the experimental data set. The remaining cohort formed our experimental data set and was categorized by using the American Diabetes Association’s guidelines [31], in which patients with HbA_1c_ readings of more than 5.7% are considered as being in the prediabetic range, while those with less than 5.7% are considered to be in the normal range.

Most medical data sets are imbalanced [32-34]. These imbalances occur when the proportion of one class of patients in the data set is greater than its counterpart class [35,36]. However, unusually, our experimental data set was not imbalanced. Slightly over half of the patients in our experimental data set (9826/18,844, 52.14%) were found to have elevated levels of HbA_1c_ (≥5.7%) while 47.86% (9018/18,844) of patients had normal HbA_1c_ levels (<5.7%). This can be ascribed to the high incidence of diabetes in the region from which the data set was collected [37].

**Figure 1:**
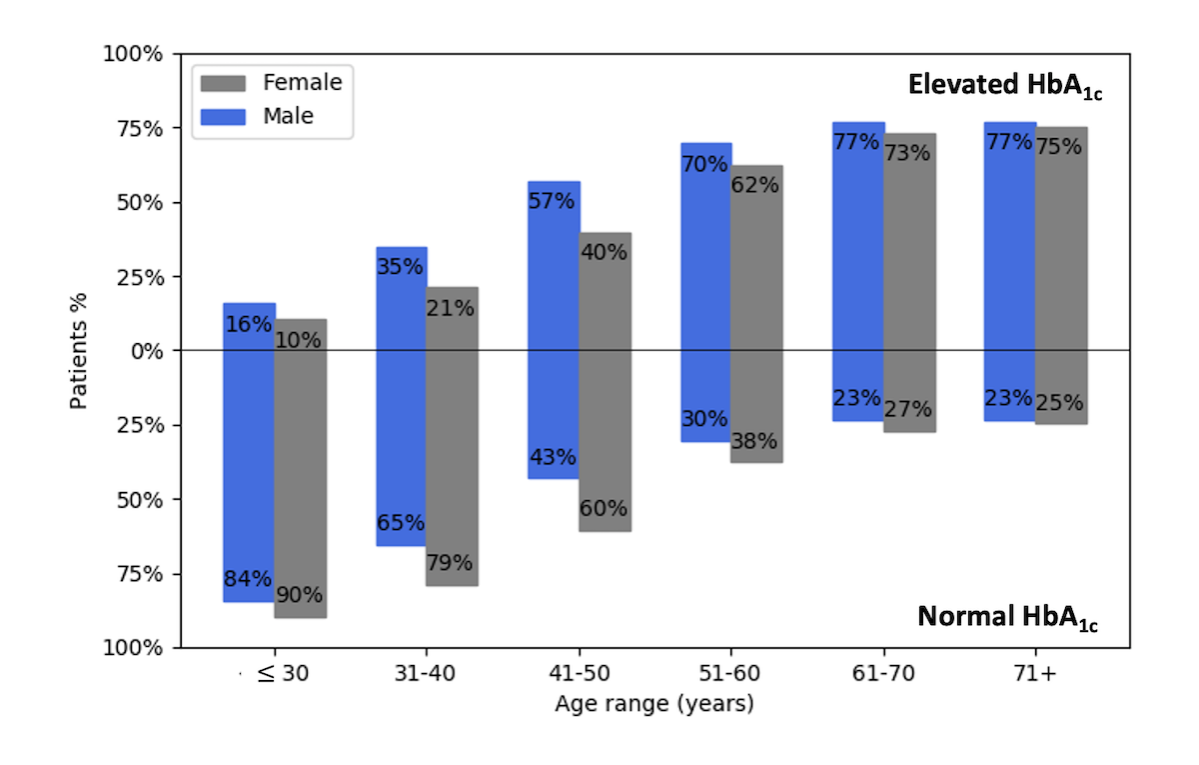
HbA_1c_ elevation levels distributed over age range and gender in the King Abdullah International Medical Research Center (KAIMRC) data set (before sampling). HbA_1c_: glycated hemoglobin.

A detailed illustration of the patients’ class distribution (HbA_1c_ levels) by age groups and gender is shown in Figure 1. This shows that as the age of patients increased, so did the proportion of patients who had elevated HbA_1c_ levels. The data set also exhibited a balanced gender distribution, with 49.40% (9308/18,844) of the patients being male and 50.60% (9536/18,844) being female. However, the proportion of male patients with elevated levels of HbA_1c_ (≥5.7%) was greater than that of the female patients. Also, female patients with normal levels of HbA_1c_ (<5.7%) made more visits than did males. Table 1 shows the profile for the distribution of HbA_1c_ elevation levels organized by gender.

**Table 1:** Profile for the class distribution over gender.

Characteristics		HbA_1c_^a^ <5.7%, n/N (%)	HbA_1c_ ≥5.7%, n/N (%)
**Number of patients** **(N=18,844)**			
	Total	9018/18,844 (47.86)	9826/18,844 (52.14)
	Male	3764/9018 (41.74)	5544/9826 (56.42)
	Female	5253/9018 (58.26)	4282/9826 (43.58)
**Number of visits (N=157,600)**			
	Total	79,607/157,600 (50.51)	77,993/157,600 (49.49)
	Male	31,620/79,607 (39.72)	41,591/77,993 (53.32)
	Female	47,987/79,607 (60.28)	36,402/77,993 (46.68)

^a^HbA_1c_: glycated hemoglobin.

### Feature Selection and Data Sampling

Six main variables (features) were extracted from the KAIMRC EHR data set to be used in this study. These features, which were selected first for their theoretical association with hyperglycemia and second for their availability in the KAIMRC data set, were the following: age, BMI, estimated glomerular filtration rate (eGFR), RBS, total cholesterol, and non–high-density lipoprotein. The lab codes of the features used are available in Multimedia Appendix 1 Table S1. The descriptive statistics (using the data for the current visit only for unique patients), units, and *P* values for the selected features are presented in Table 2.

**Table 2:** Descriptive statistics of the selected features from the King Abdullah International Medical Research Center (KAIMRC) data set.

Feature	HbA_1c_^a^ 5.7%, mean (SD)	HbA_1c_ 5.7%, mean (SD)	*P* value
Age (years)	43.94 (16.38)	58.92 (15.12)	<0.001
BMI (Kg/m^2^)	29.11 (6.75)	30.90 (6.55)	<0.001
eGFR^b^ (ml/min/1.73 m^2^)	100.03 (29.22)	85.81 (28.239)	<0.001
RBS^c^ (mmol/L)	5.45 (1.26)	7.88 (4.19)	<0.001
CHOL^d^ mean (mmol/L)	4.65 (1.07)	4.42 (1.20)	<0.001
non-HDL^e^ mean (mmol/L)	3.45 (1.01)	3.37 (1.115)	<0.001

^a^HbA_1c_: glycated hemoglobin.

^b^eFGR: estimated glomerular filtration rate.

^c^RBS: random blood sugar.

^d^CHOL: total cholesterol.

^e^non-HDL: non–high-density lipoprotein.

It is very common in clinical practice that physicians may require some lab tests and vital signs to be frequently recorded. In these cases, the average value of all readings taken on a given day (the basic time interval used for this study) was used. For inpatient visits, only data for the first day were considered, and, where there were missing values, the first available values from the visit were used.

**Figure 2:**
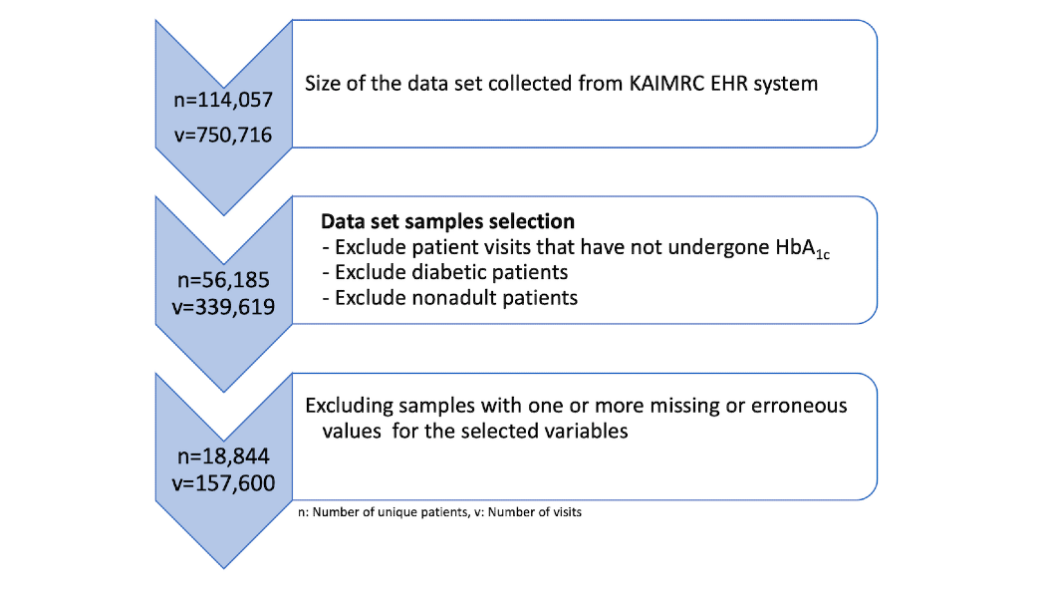
Details of the sampling approach performed on the KAIMRC data set. EHR: electronic health record; HbA_1c_: glycated haemoglobin; KAIMRC: King Abdullah International Medical Research Center.

For the purpose of this study, we aimed at predicting the HbA_1c_ levels (≥5.7%) for current (last) patient visits only. Unlike the sampling approach used by Wells et al, which was based on independent hospital visits for patients (including for the same patients), the sampling approach used in this study included independent patients to ensure only unseen patients data were used for testing the models. Although we aimed to identify patients with elevated levels of HbA_1c_ from a nondiabetic population, patients previously diagnosed with diabetes were excluded. We also excluded nonadult patients and those with erroneous or missing values [24]. Figure 2 shows the details of the tasks performed to refine the sample selection. This resulted in a reduction in the size of the experimental data set from 114,057 patients with 750,709 visits to 18,844 unique patients with 157,600 visits.

The inputs (input features space) for the models used in this study were continuous values. Values for age, eGFR, RBS and total cholesterol features were directly available in the KAIMRC data set. The values for the BMI and non–high-density lipoprotein variables were calculated from other available features using the formulae in Multimedia Appendix 2.

### Input Preparation for the Models

The input structure for the deep learning model was organized as a matrix, based on current and previous time-stamped patient visits. It contained the current visit data concatenated with approximated values for the selected features from all previous visits, which we refer to as the “Approximated Time Series Data”.

Each patient visit was described by the selected features, represented as *x*_1_, *x*_2_ …, *x_n_*. These features were formed as episodes based on the time-stamped values available in each visit (v_i_).

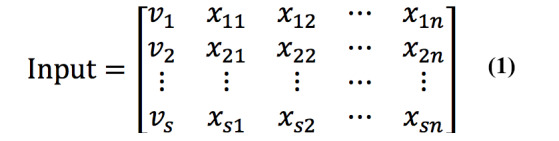



Here, *x_ij_* is the feature value at a patient visit (0 < *i* ≥ *s*, 0 < *j* ≥ *n*); *s* is the number of time series steps (the length of the input sequence); and *n* is the number of features for each time step, which was set to 6 as explained earlier.

If the number of visits (longitudinal time series visits) for a patient was fewer than *s*, the input for this patient was padded out with the mean value of the available visits to compensate for the missing time series data (Multimedia Appendix 3 shows an example of the padding approach used). Where the number of longitudinal visits for a patient was more than *s*, the piecewise aggregation approximation (PAA) technique [38] was applied to the data for these visits to account for all data from patient visits.

PAA transforms the longitudinal time series data using *s* as a number of sliding windows (or segments) into a reduced number of time steps data (approximated) employing the mean value of the series falling within that window (segment) [39]. We tested the models with several values for the size of the sliding window (*s*), and 3 was shown to be the optimal value. The formula used to calculate the approximated time-series data was as follows:


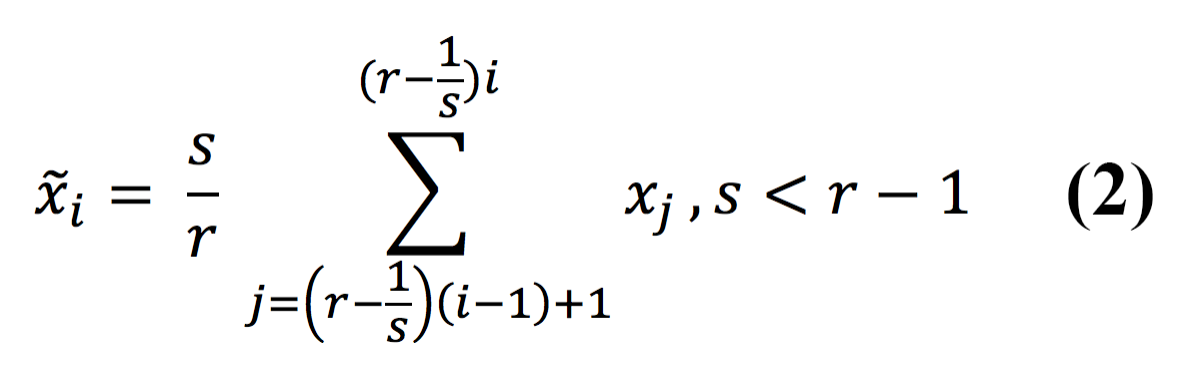


Where 
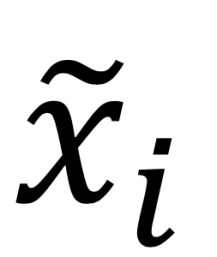
 represents the approximated value for *x*, *r* is the total number of visits for a patient, and *s* is the reduced number of time series steps (Multimedia Appendix 4 shows an example of the PAA technique used).

The approximated time series data forming the output of the PAA was then concatenated with the current visit data to form the final input for the deep learning model. As the MLR, RF, SVM, and LR models are not capable of handling multidimensional data (formed as matrices), the output of the PAA was reorganized for these into a single-dimensional input by vectorizing the matrix used in equation 1 as below:

Input = [*x*_11_*x*_12_*x*_13 …_*x_sn_*]    **(3)**

The last data preprocessing task before training the predictive models was data scaling. The experimental data set was scaled using the normalization technique that rescales the ranges of each of the features to be between 0 and 1 using minimum and maximum values of that feature.

### Predictive Models and Experimental Setups

As a baseline comparison, we employed the MLR model used by Wells et al [21], and compared the results from this with those from 4 commonly used machine learning models.

The MLR model is used to create a mathematical equation that can best calculate the probability of a value by assigning weights (coefficients) to the independent variables (features) based on their importance [40]. In this study we employed the same approach used by Wells et al by which the continuous features were fitted into the MLR model using restricted cubic splines technique with 3 knots. When we used the longitudinal input, the variables that caused collinearity were excluded.

Random forest is an algorithm very commonly used for classification. It combines several decision trees that are generated during the training process. Each decision tree is trained using a random subset of the training data set. The final classification is then based on the majority voting results of all generated decision trees [41]. The quality function used in the employed RF model is the Gini importance, with a value of 100 for the number of tree parameters.

Logistic regression is commonly used to solve binary classification problems. It calculates the odds ratio of the variables and is similar to MLR but uses a binomial distribution of the dependent variable (ie, more than 1). Thus, it includes a logit function that handles different types of relationships between the dependent and independent variables [42,43].

Support vector machine was introduced by Vapnik [44] in 1998. It can solve both classification and regression problems. It uses the training feature space to decide on the separation boundaries (hyperplane) that best divides the training data set into regions, 1 for each class. The very close points to the hyperplanes are the support vectors. SVMs also use kernels to help enhance class separation by mapping the training features into a higher dimensional space with an increased number of dimensions [44,45]. The kernel function used in the SVM model employed is a radial base function with a value of 1 for the cost parameter (*C*).

A multilayer perceptron, also known as a feed-forward neural network, is one of the most common deep learning approaches. It is mainly used to address supervised learning problems by learning the dependencies between the input layer (the features or variables) and output layer (the classification decision) using a fully connected hidden layer in between. The layers, including hidden ones, contain a number of neurons that are connected to the neurons of the next and previous layers via weights and nonlinear functions. MLP uses a backpropagation algorithm to update the weights and biases within the hidden layers to minimize the output error rate [25,46].

**Figure 3:**
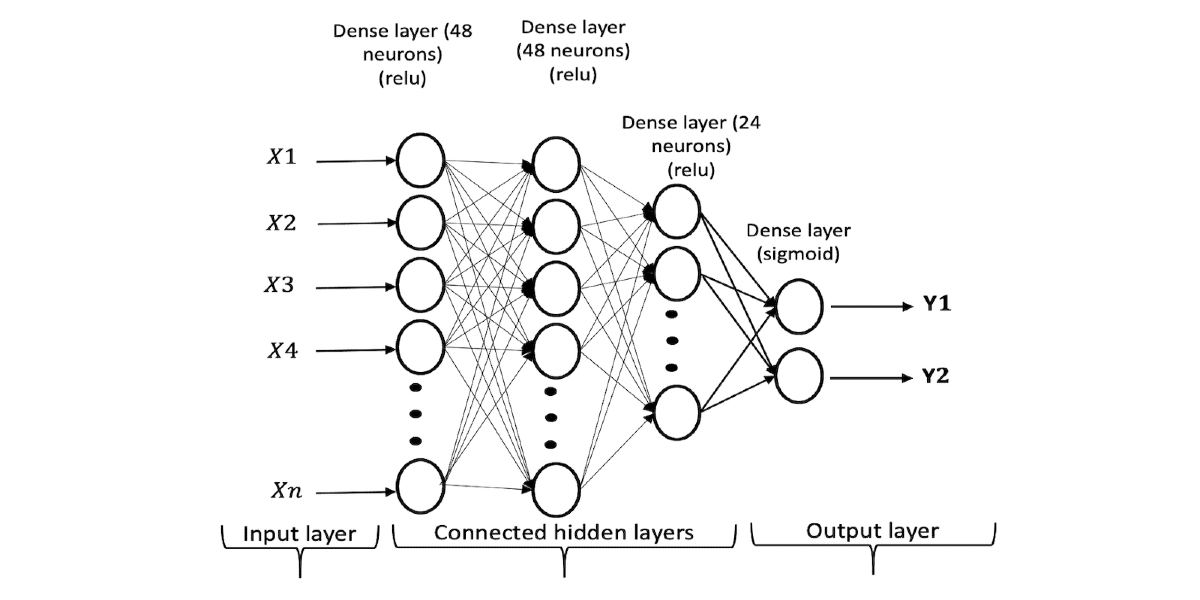
The structure used for multilayer perceptron trained with the longitudinal data. relu: rectified linear unit.

To optimize the MLP model, fine-tuning of the structure and hyperparameters was performed and involved the number of hidden layers and neurons, activation functions, optimizers, and loss functions. The optimized structure of the MLP model used in this study contained 3 hidden layers. The number of neurons in the hidden layers were 48, 48, and 24, respectively. The final layer (the output layer) contained 2 neurons for the final output of the model (*Y*1 for normal HbA_1c_ or *Y*2 for elevated HbA_1c_). A rectified linear unit activation function was used in the 3 hidden layers, while a sigmoid was used in the output layer. The detailed structure of the MLP model is shown in Figure 3. The model was trained using an Adam optimizer with mean squared error as the loss function.

### Evaluation of Model Performance

The models all employed the same data preprocessing, training, and testing techniques. The models were validated using the 10-fold cross-validation technique. The k-fold cross-validation is one of the most commonly used approximation approaches for validating the obtained results [47,48]. For the MLP model, 100 epochs were used to train each fold.

As our measure for evaluating and comparing the performance of the proposed models, we used the area under the receiver operating characteristic (AUC-ROC) curve, which is equal to the concordance statistic [49]. We also report values for a set of measures that are commonly used in clinical applications: balanced accuracy (that calculates the recall average for each class), overall accuracy, *F* score, precision, and precision-recall area under the curve (PR-AUC).

To determine the importance that the black box models (SVM and MLP) place upon each variable, we first computed the SHAP values and LIME scores for all samples in our data set and then calculated the average absolute SHAP value and LIME score for each predictor.

## Results

Table 3 shows the performance metrics obtained using the MLR, RF, SVM, LR, and MLP models with and without the longitudinal data. The results show that the models achieved competitive performance using the reported measures. The LR and MLP models trained with and without the longitudinal data achieved better performance with regards to the AUC-ROC measure than did the MLR (statistical model employed by Wells et al) or the RF and SVM models (more details about AUC-ROC and PR-AUC curve plots are presented in Multimedia Appendix 5). The results also show that the SVM, LR, and MLP models trained with and without the longitudinal data achieved better performance than did the MLR and RF models using the balanced accuracy measure.

Table 3 also shows that all models, including the MLR, achieved better performance using all reported measures when they were trained with the features from patients’ longitudinal data. The MLP with longitudinal data slightly outperformed all other models with respect to the reported measures.

**Table 3:** Classifiers performance for current glycated hemoglobin level prediction.

Model			AUC-ROC^a^, % (SD)		Balanced accuracy, % (SD)		Accuracy, % (SD)		*F* score, % (SD)		Precision, % (SD)		PR-AUC^b^, % (SD)
**MLR^c^**													
	No^d^	81.38 (3.82)		72.74 (4.15)		73.59 (3.79)		74.91 (5.12)		73.20 (5.05)		82.14 (6.04)	
	Yes^e^	82.45 (4.09)		73.49 (4.19)		74.30 (4.02)		75.11 (6.00)		74.36 (5.26)		83.45 (6.29)	
**RF^f^**													
	No	80.82 (1.14)		72.57 (1.17)		72.64 (1.14)		73.97 (1.04)		73.42 (1.84)		82.03 (1.35)	
	Yes	82.38 (1.04)		73.86 (0.98)		73.91 (0.95)		75.07 (0.86)		74.81 (1.68)		84.06 (1.17)	
**SVM^g^**													
	No	81.05 (1.04)		73.69 (1.35)		73.88 (1.33)		75.76 (1.18)		73.42 (1.90)		80.56 (1.48)	
	Yes	82.04 (0.89)		74.25 (1.11)		74.40 (1.08)		76.08 (0.92)		74.20 (1.65)		83.16 (1.19)	
**LR^h^**													
	No	81.51 (1.26)		73.18 (1.10)		73.17 (1.08)		73.96 (1.03)		74.88 (1.69)		82.49 (1.46)	
	Yes	82.59 (1.04)		74.11 (1.15)		74.05 (1.13)		74.55 (0.98)		76.31 (1.72)		84.13 (1.04)	
**MLP^i^**													
	No	82.07 (1.06)		73.61 (1.04)		73.83 (1.03)		75.87 (1.10)		73.07 (1.62)		83.42 (1.19)	
	Yes	83.22 (0.92)		74.45 (1.18)		74.55 (1.18)		75.99 (1.95)		74.78 (2.07)		84.85 (0.78)	

^a^AUC-ROC: area under the receiver operating characteristic.

^b^PR-AUC: precision-recall area under the curve.

^c^MLR: multiple logistic regression.

^d^Without longitudinal data.

^e^With longitudinal data.

^f^RF: random forest.

^g^SVM: support vector machine.

^h^LR: logistic regression.

^i^MLP: multilayer perceptron.

**Figure 4:**
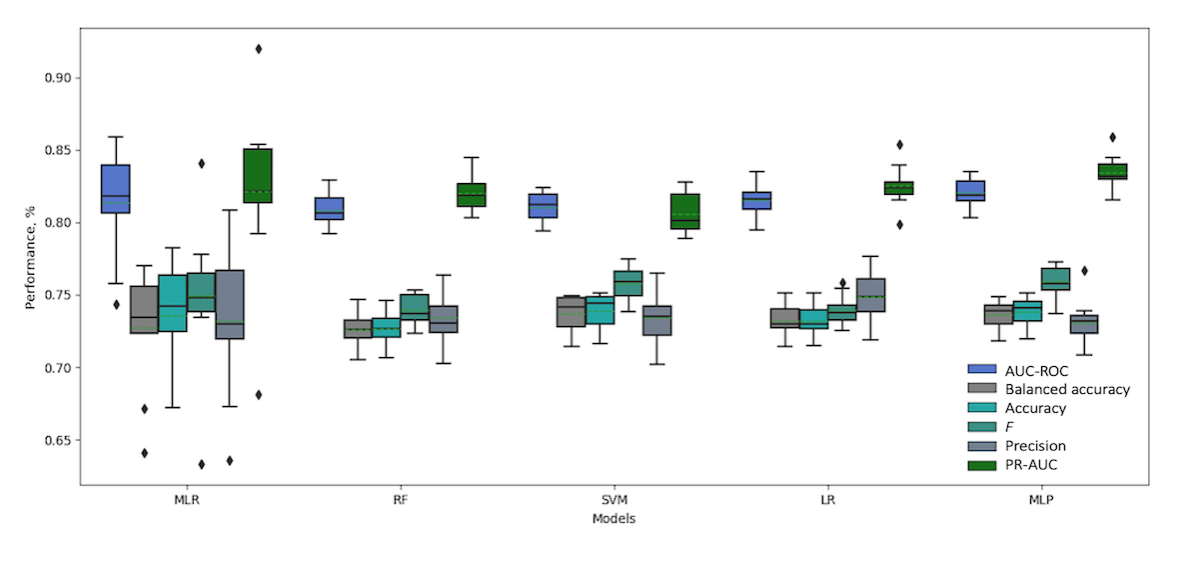
Box plot showing the detailed 10-fold performance of all models trained without longitudinal data. AUR-ROC: area under the receiver operating characteristic; LR: logistic regression; MLP: multilayer perceptron; MLR: multiple logistic regression; PR-AUC: precision-recall area under the curve; RF: random forest; SVM: support vector machine.

**Figure 5:**
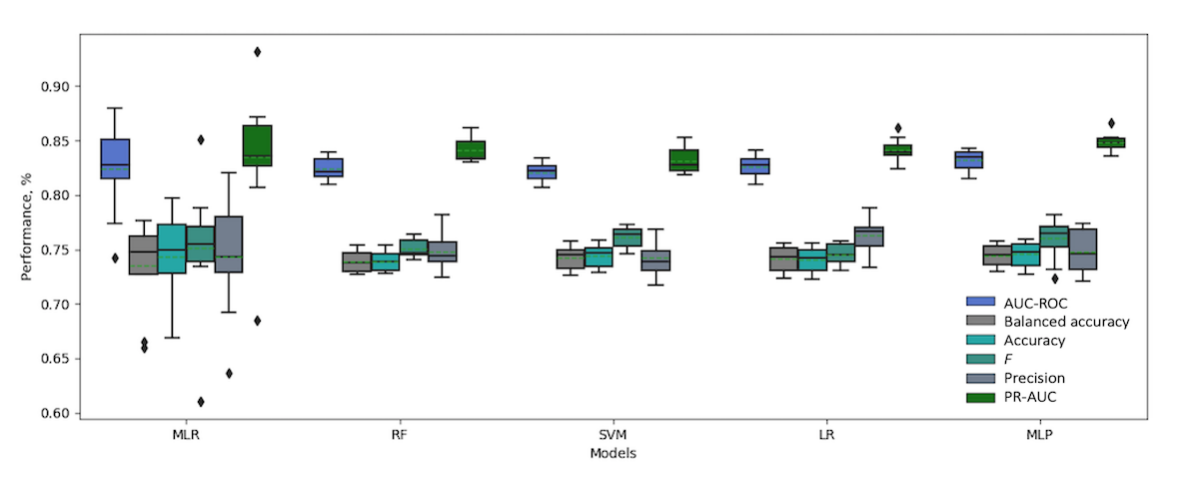
Boxplot showing the detailed 10-fold performance of all models trained with longitudinal data. AUR-ROC: area under the receiver operating characteristic; LR: logistic regression; MLP: multilayer perceptron; MLR: multiple logistic regression; PR-AUC: precision-recall area under the curve; RF: random forest; SVM: support vector machine.

Figure 4 summarizes the 10-fold performance achieved for the set of measures where the models were trained without longitudinal data, and Figure 5 shows the performance where they were trained with the longitudinal data. Both figures show a more consistent prediction trend for RF, LR, SVM, and MLP with and without longitudinal data, as the measures for these models show a small variation between the folds. As shown in Figure 4 and Figure 5, the SD values for MLR with and without longitudinal data are larger than those for the other models. This indicates that the machine learning models used can not only enhance the performance, but can also improve the classification confidence for HbA_1c_ prediction.

Table 4 shows the ranked order of importance of the set of predictors used for training the models. Further details on the actual importance values for each model are provided in Multimedia Appendix 6 (refer to Multimedia Appendix 7 for more details of the MLR and LR calculator). Calculating the importance of the predictors for the MLR models using vectorized longitudinal data was not possible due to the collinearity caused by having multiple variables for BMI. The order of importance results obtained using the SHAP method for both the SVM and MLP were identical to those obtained using LIME and provided greater confidence in the explainable methods used (see Multimedia Appendix 6).

**Table 4:** Order of importance of predictors for the models.

Model		Importance rank					
		1st	2nd	3rd	4th	5th	6th
**MLR^a^**							
	No^b^	Age	RBS^c^	BMI	CHOL^d^	Non-HDL^e^	eGFR^f^
**RF^g^**							
	No	Age	RBS	BMI	eGFR	CHOL	Non-HDL^h^
	Yes^h^	RBS	Age	CHOL	eGFR	Non-HDL	BMI
**LR^i^**							
	No	RBS	Age	Non-HDL	CHOL	BMI	eGFR
	Yes	RBS	Age	Non-HDL	eGFR	CHOL	BMI
**SVM^j^ (SHAP^k^ & LIME^l^)**							
	No	Age	RBS	BMI	Non-HDL	CHOL	eGFR
	Yes	RBS	Age	CHOL	Non-HDL	BMI	eGFR
**MLP^m^ (SHAP & LIME)**							
	No	RBS	Age	Non-HDL	CHOL	BMI	eGFR
	Yes	RBS	Age	eGFR	CHOL	Non-HDL	BMI

^a^MLR: multiple logistic regression.

^b^Without longitudinal data.

^c^RBS: random blood sugar.

^d^CHOL: total cholesterol.

^e^non-HDL: non–high-density lipoprotein.

^f^eGFR: estimated glomerular filtration rate.

^g^RF: random forest.

^h^With longitudinal data.

^i^LR: logistic regression.

^j^SVM: support vector machine.

^k^SHAP: Shapley Additive Explanations.

^l^LIME: local interpretable model-agnostic explanations.

^m^MLP: multilayer perceptron.

**Figure 6:**
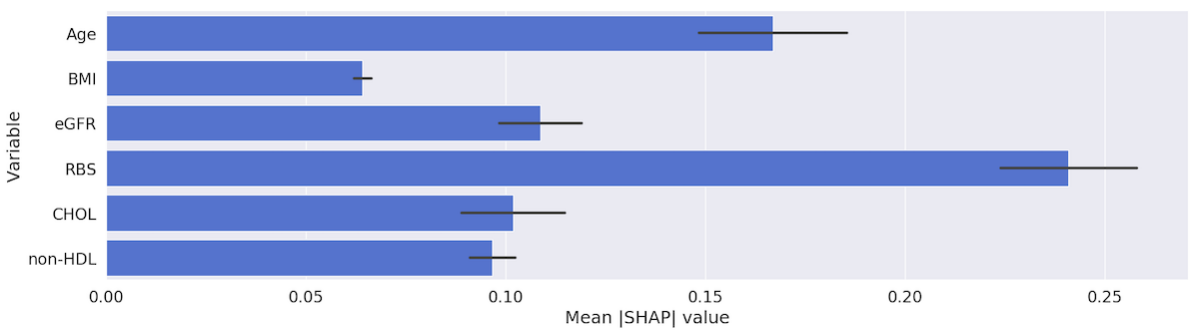
Relative importance of predictors obtained from the multilayer perceptron trained with longitudinal data using SHAP. CHOL: total cholesterol; eGFR: estimated glomerular filtration rate; non-HDL: non–high-density lipoprotein; RBS: random blood sugar; SHAP: Shapley Additive Explanations.

**Figure 7:**
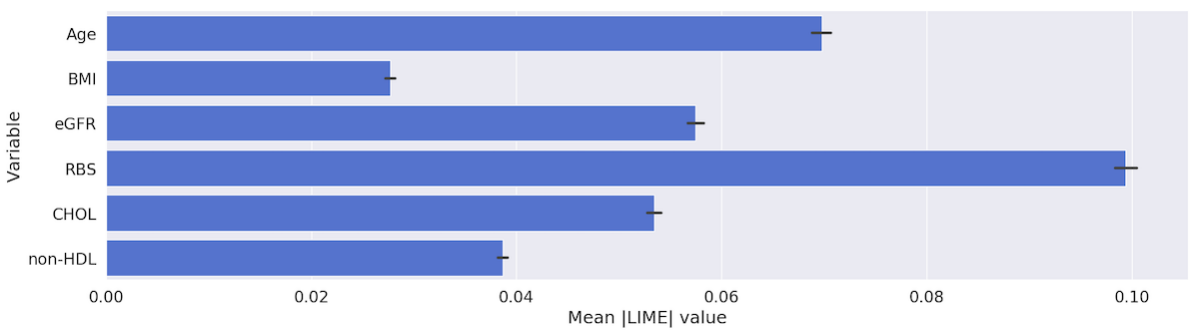
Relative importance of predictors obtained from multilayer perceptron trained with longitudinal data using LIME. CHOL: total cholesterol; eGFR: estimated glomerular filtration rate; LIME: local interpretable model-agnostic explanations; non-HDL: non–high-density lipoprotein; RBS: random blood sugar.

Table 4 and the figures in Multimedia Appendix 6 show that all of the models were heavily and interchangeably reliant on age and RBS when making classification decisions. The RF and SVM models, when trained with longitudinal data, ranked RBS over age. Figure 6 and Figure 7 highlight the importance that our best performing model, MLP, placed upon the features in our data set using SHAP and LIME, respectively. Both figures show that the RBS contributed the most to the MLP’s final prediction, while the patient’s BMI contributed the least.

For all models trained with longitudinal data, BMI was ranked lower than when the models were trained without longitudinal data. However, the importance value produced for the BMI variable from the models was still not insignificant (see the figures in Multimedia Appendix 7). This indicates that models are able to find subtle relationships in the longitudinal data that are more relevant to the prediction than is BMI, rendering it less important.

When MLP and LR models trained on the longitudinal data were used, the eGFR variable was ranked higher than total cholesterol and BMI, in contrast to when these were trained on the current visit only. None of the other models trained with the current visit only, except for RF, considered it important. Again, we ascribe this to the information that the model learns from the variations of eGFR values between a patient’s visits (longitudinal EHR data).

**Figure 8:**
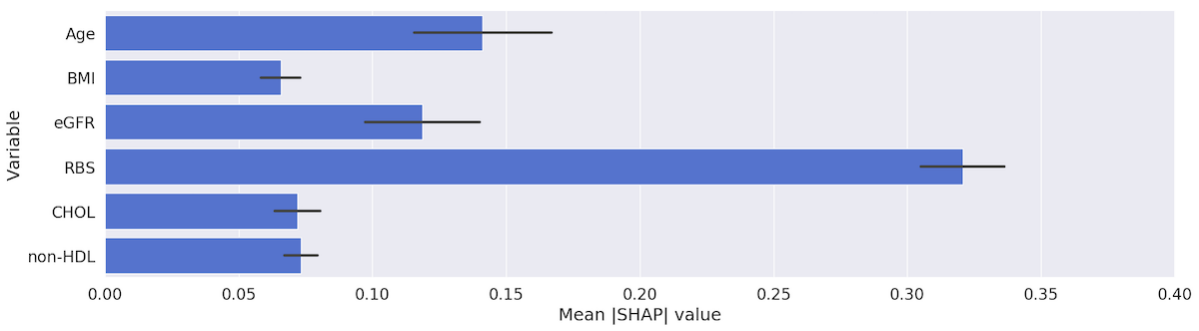
An example showing the SHAP values for a randomly selected sample with elevated glycated hemoglobin levels (≥5.7%). CHOL: total cholesterol; eGFR: estimated glomerular filtration rate; non-HDL: non–high-density lipoprotein; RBS: random blood sugar; SHAP: Shapley Additive Explanations.

**Figure 9:**
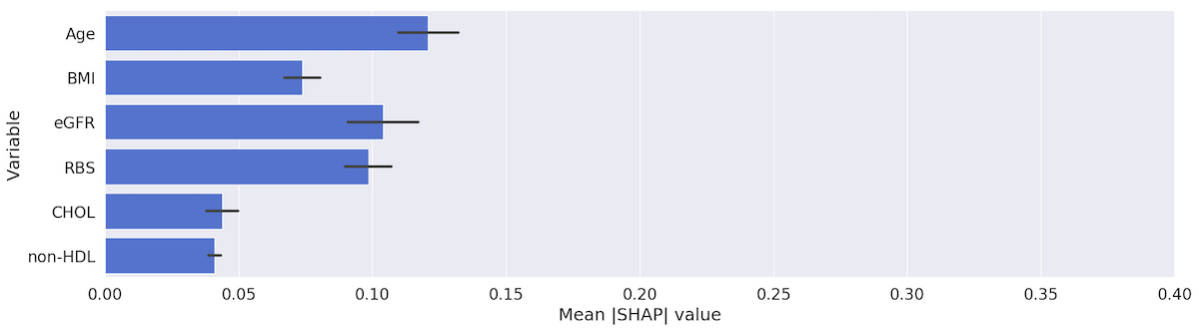
An example showing the SHAP values for randomly selected sample with normal glycated hemoglobin levels (<5.7%). CHOL: total cholesterol; eGFR: estimated glomerular filtration rate; non-HDL: non–high-density lipoprotein; RBS: random blood sugar; SHAP: Shapley Additive Explanations.

SHAP values are calculated on the sample level. Figures 8 and 9 illustrate the SHAP values for 2 randomly selected sample patients from our data set. These figures highlight how different inputs have different SHAP values. The patient in Figure 8 (for whom our model correctly predicted elevated HbA_1c_ levels of ≥5.7%) had a higher RBS value than did the patient in Figure 9 (for whom our model correctly predicted normal HbA_1c_ levels of <5.7%). This explains why our MLP model placed much more importance on the RBS value of the patient in Figure 6.

**Figure 10:**
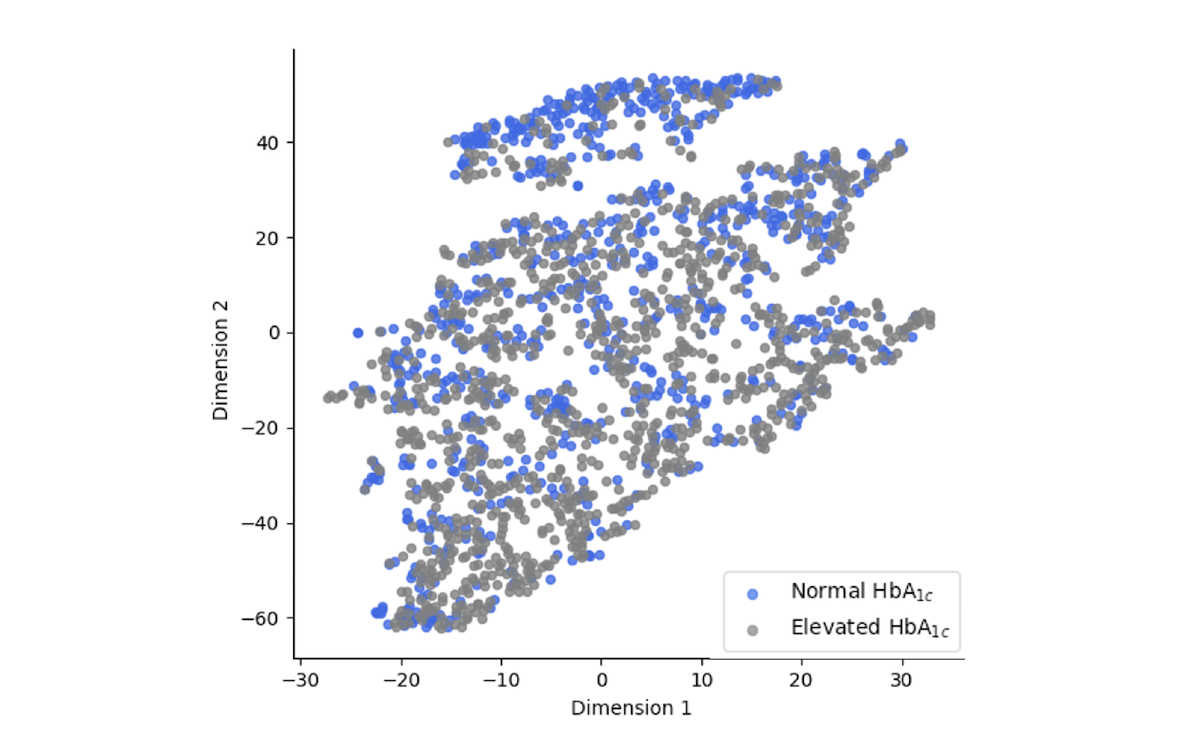
Two-dimensional visualization using t-distributed stochastic neighbor embedding for a randomly selected subset of the data. HbA_1c_: glycated hemoglobin.

The task of predicting HbA_1c_ elevation risk can be challenging. Figure 10 provides a visualization of the data points for the 2 classes (prediabetic with ≥5.7%; normal with <5.7%) after mapping of the data points (for the test data) into 2 dimensions with t-distributed stochastic neighbor embedding was performed [50]. The overlap in the data points visualized in the figure demonstrates the challenge of separating the patients with and without elevated levels of HbA_1c_ (≥5.7%) in the KAIMRC data set. We avoided intensive feature engineering techniques in the sampling approach used. However, the approaches adopted were able to achieve promising results with an accuracy of 83.22% for the AUC-ROC using MLP with historical data.

In summary, all models showed promising results for predicting the current HbA_1c_ elevation levels (≥5.7%) with EHR data. The results emphasize that the HbA_1c_ predictive models can exhibit more learnability when they are trained with the longitudinal patient data observations typically available from EHR systems.

## Discussion

### Strengths and Limitations

EHR systems were adopted for the purpose of improving health care outcomes and were not originally intended for research purposes [19]. Patient data stored in EHR systems can be obtained at irregular intervals, as lab instructions are carried out with different frequencies based on the physician's decisions and a patient’s visit patterns. It is very common that medical data extracted from EHR systems suffer from problems such as irregularity, incompleteness, and noisy and imbalanced data [13]. These can be challenging obstacles for any technology used for predictive analytics.

In our study, the sampling approach used did not affect the balanced nature of the data set used. As shown in Figure 2, there were 56,185 unique patients present before removal of the records with 1 or more missing values. The number of unique patients with elevated HbA_1c_ levels (≥5.7%) before removal of the incomplete records was 27,354, resulting in a retention of 48.68% (27,354/56,185). The number of unique patients with normal HbA_1c_ levels was 28,831, resulting in a retention of 51.32% (28,831/56,185). We would argue that the absence or the presence of the HbA_1c_ readings is not random, as the sample was collected from the population of Saudi Arabia and thus the likelihood of a patient taking an HbA_1c_ test is large because of the prevalence of diabetes in this country [51]. This may affect the reproducibility of this work using different populations from different countries especially those with lower rates of diabetes.

**Figure 11:**
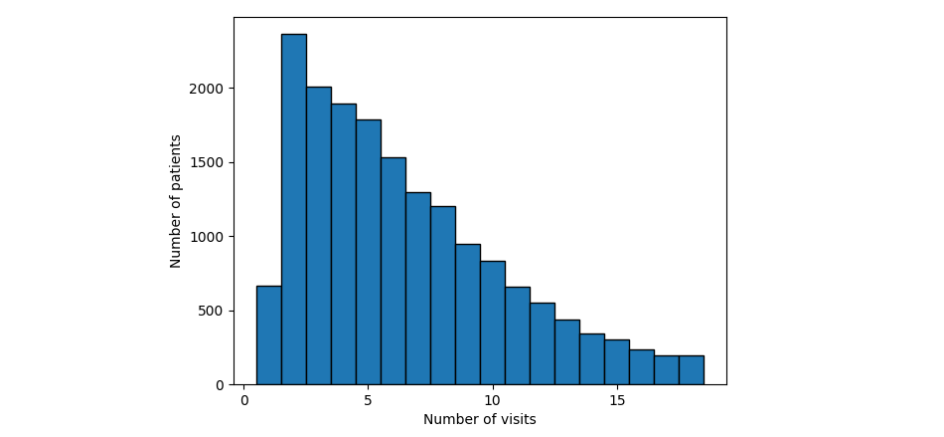
Histogram showing the trend in the number of visits made by patients.

**Figure 12:**
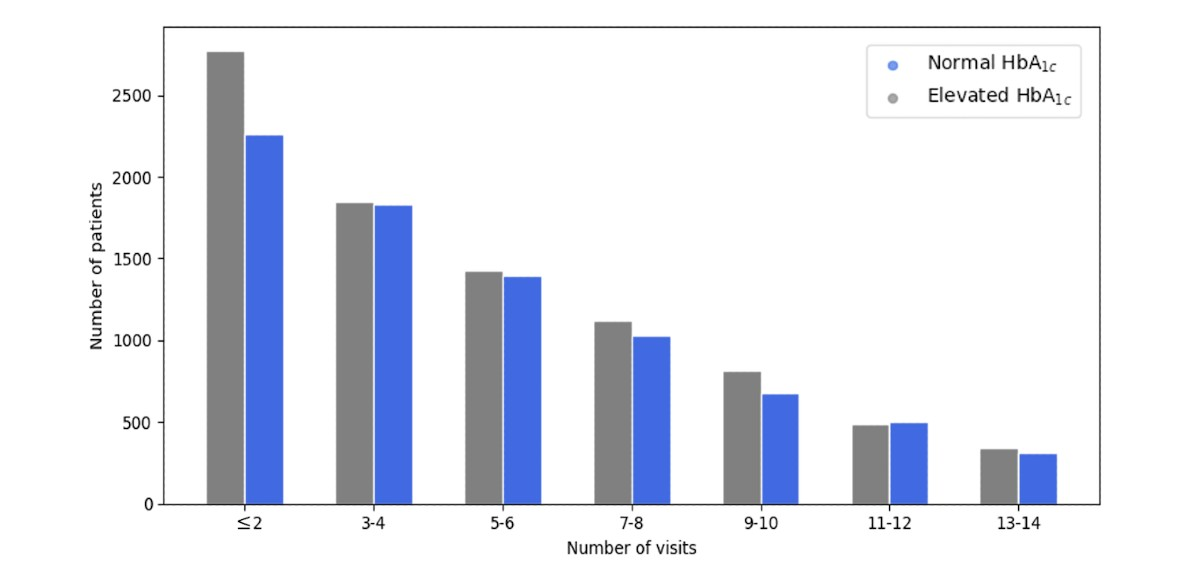
The details for the number of visits made over number of patients. HbA_1c_: glycated hemoglobin.

It is hoped that these outcomes will encourage further investigation into the predictability of current HbA_1c_ levels (≥5.7%) using more of the readings normally provided in EHR data. For example, other important readings such as FBS and triglycerides have shown clinical correlations with diabetes [52]. In addition, our data set contained only 3 years of patient data, which limits the number of patient visits recorded. Figure 11 shows the number of visits made by patients from 2016 to 2018, while Figure 12 details the number of visits made by patients (after removal of the outliers) over HbA_1c_ levels. Both figures show that the majority of the patients have made relatively few visits: 52% (8713/16818) of the patients made 4 visits or fewer over the 3 years (1.3 visit per year). This also justifies the size of the sliding window (*s*=3) as the optimal input size for the models used. However, we hypothesize that the longitudinal behavior of the features used can be enriched by including more values obtained over longer periods. Therefore, incorporating more features and their longitudinal behavior over longer periods into the models used in this study would likely improve the prediction performance of our chosen models.

Variations in the data or model produce slightly different attribution values. However, due to the critical nature of many health care applications, it is always important to verify that the models make “sensible” predictions. Without the use of SHAP/LIME, this would be hard to verify for any nonlinear model. Although it is possible to see that the models have high performance, we would be unable to verify that a model is not making spurious correlations. Furthermore, through the use of SHAP, we can verify that MLPs trained on the longitudinal data are learning to use the extra information contained in the longitudinal data (as indicated by the higher importance of eGFR), allowing us to pinpoint the reason these models gain higher performance.

To investigate the effect of temporal dependencies in the data, this study investigated the use of other deep learning models along with the MLP, including long short-term memory (LSTM) and bidirectional LSTM [25,53] for HbA_1c_ prediction. Table 5 reports the results of using these models. The MLP model achieved similar performance to the LSTM and bidirectional LSTM models according to all reported measures. This suggests that directly modeling the temporal dynamics in the data is not very helpful. This could be due to the short lengths of the time series or a too-weak temporal dependency.

**Table 5:** LSTM and BiLSTM Classifiers performance trained with longitudinal data for current HbA_1c_ levels prediction.

Model	AUC-ROC^a^, % (SD)	Balanced Accuracy, % (SD)	Accuracy, % (SD)	*F* score, % (SD)	Precision, % (SD)	PR-AUC^b^, % (SD)
LSTM^c^	83.26% (0.91)	74.17% (1.05)	74.59% (1.23)	75.64% (1.50)	74.59% (3.26)	81.88% (0.95)
BiLSTM^d^	83.16% (0.87)	74.21% (1.24)	74.30% (1.15)	75.46% (1.39)	75.19% (2.36)	84.75% (0.75)

^a^AUC-ROC: area under the receiver operating characteristic.

^b^PR-AUC: precision-recall area under the curve.

^c^LSTM: long short-term memory.

^d^BiLSTM: bidirectional LSTM.

Generalizing our findings using other data sets is challenging because of the accessibility and privacy restrictions that apply to medical data sets. For this reason, and because of the lack of similar studies that have used machine learning for HbA_1c_ prediction with EHR data, comparing the performance achieved by the models outlined in this study with those developed by other researchers will require the availability of alternative anonymized data sets.

### Conclusions

We believe that this study is the first to investigate the performance of machine learning models used with EHR data for predicting current HbA_1c_ elevation risk (≥5.7%) for nondiabetic patients. It is also the first to investigate employing the longitudinal data that are normally stored on EHR systems to enhance the prediction of HbA_1c_ elevation levels. Our findings show that the MLP model achieves better results when a patient’s longitudinal data are combined with current visit data, and the use of longitudinal data also affects the relative importance for the predictors used.

As this work formed a continuation of previous work [24], we avoided changing the sampling approach used. However, studying the impact of applying different sampling approaches could be valuable to explore in future work as would the use of a larger data set with more variables and the recording of longitudinal behavior over longer periods.

## Appendix

Multimedia Appendix 1 Lab test and diagnostic codes.

Multimedia Appendix 2 Formulae for the calculated variables.

Multimedia Appendix 3 An example of the padding approach used.

Multimedia Appendix 4 An example of the PAA technique.

Multimedia Appendix 5 AUC-ROC and PR-AUC curves for the models (with 10 folds) trained with longitudinal data.

Multimedia Appendix 6 Variable relative importance charts for the models.

Multimedia Appendix 7 Multiple logistic regression (MLR) and logistic regression (LR) details.

## Abbreviations

AUR-ROC: area under the receiver operating characteristic
eGFR: estimated glomerular filtration rate
EHR: electronic health records
FBS: fasting blood sugar
HbA_1c_: glycated hemoglobin
KAIMRC: King Abdullah International Medical Research Center
LIME: local interpretable model-agnostic explanations
LR: logistic regression.
LSTM: long short-term memory
MLP: multilayer perceptron
MLR: multiple logistic regression
PAA: piecewise aggregation approximation
PR-AUC: precision-recall area under the curve
RBS: random blood sugar
RF: random forest
SHAP: Shapley Additive Explanations
SVM: support vector machine
T2DM: type-2 diabetes mellitus
WHO: World Health Organization
