# Supplementary material for: Improving Current Glycated Hemoglobin Prediction in Adults: Use of Machine Learning Algorithms With Electronic Health Records
Source: JMIR Med Inform. 2021 May 24;9(5):e25237. doi: 10.2196/25237 (PMC8185616; doi:10.2196/25237)
Supplement: Multimedia Appendix 1 [file medinform_v9i5e25237_app1.pdf]

Multimedia Appendix 1

Lab test and diagnostic codes

Table S1. Lab test codes used by KAIMRC.

Lab Test Code	Description	Units used
L3000002	estimated Glomerular Filtration Rate (eGFR)	mL/min/1.73 m^2
L3900229	Random Blood Sugar (Glucose) Level (RBS)	mmol/L
L3000026	Low Density Lipoprotien (LDL)	mmol/L
L3000006	Total Cholesterol (CHOL)	mmol/L
L3000013	High Density Lipoprotien (HDL)	mmol/L

Table S2. ICD10 Hyperglycemia diagnostic codes used by KAIMRC.

Diagnostic Code	Description
E11	Type 2 Diabetes Mellitus
E14	Diabetes Mellitus
E10	Type 1 Diabetes Mellitus
E139	Familial Diabetes Mellitus
R73	Hyperglycemia
O24	Gestational diabetes
