# Supplementary material for: Improving Current Glycated Hemoglobin Prediction in Adults: Use of Machine Learning Algorithms With Electronic Health Records
Source: JMIR Med Inform. 2021 May 24;9(5):e25237. doi: 10.2196/25237 (PMC8185616; doi:10.2196/25237)
Supplement: Multimedia Appendix 2 [file medinform_v9i5e25237_app2.pdf]

Multimedia Appendix 2

Formulae for the calculated variables*:

Non-HDL Cholesterol = Total Cholesterol – HDL Cholesterol

Body Mass Index (BMI) = Weight(kg) / $(\text{Height(m)})^2$

*Reference:
https://www.whittington.nhs.uk/document.ashx?id=10724
https://www.thecalculatorsite.com/articles/health/bmi-formula-for-bmi-calculations.php
