# Supplementary material for: Improving Current Glycated Hemoglobin Prediction in Adults: Use of Machine Learning Algorithms With Electronic Health Records
Source: JMIR Med Inform. 2021 May 24;9(5):e25237. doi: 10.2196/25237 (PMC8185616; doi:10.2196/25237)
Supplement: Multimedia Appendix 3 [file medinform_v9i5e25237_app3.pdf]

Multimedia Appendix 3

Below figure shows an example of the padding approach used (In the figure, we used Cholesterol (x_1) as an example feature to demonstrate the padding approach).

Patient	Visit	Date	x_1
P1	V_2	26/12/2017	3.81
	V_2	1/11/2016	5.32

⇩

Padding the missing time-series data
with the mean

⇩

Patient	Time step	$\bar{x}_1$
P1	TS1 (current)	3.81
	TS2	5.32
	TS3	4.57
	TS4	4.57

Example of input padding when number of patient longitudinal visits is fewer than s.
