# Supplementary material for: Improving Current Glycated Hemoglobin Prediction in Adults: Use of Machine Learning Algorithms With Electronic Health Records
Source: JMIR Med Inform. 2021 May 24;9(5):e25237. doi: 10.2196/25237 (PMC8185616; doi:10.2196/25237)
Supplement: Multimedia Appendix 4 [file medinform_v9i5e25237_app4.pdf]

# Multimedia Appendix 4

Below figure shows an example of the PAA technique using a sliding window of $s = 3$ for Cholesterol ($x_1$) feature for a patient with current visit and six time-stamped visits that were available in the EHR longitudinal data for the given patient ($r = 7$).

| Patient | Visit | Date | $x_1$ |
|---|---|---|---|
| P2 | V_1 | 23/12/2018 | 4.72 |
| | V_2 | 5/7/2018 | 4.93 |
| | V_3 | 31/1/2018 | 5.94 |
| | V_4 | 20/9/2017 | 5.13 |
| | V_5 | 23/4/2017 | 6.55 |
| | V_6 | 2/10/2016 | 4.66 |
| | V_7 | 9/5/2016 | 5 |

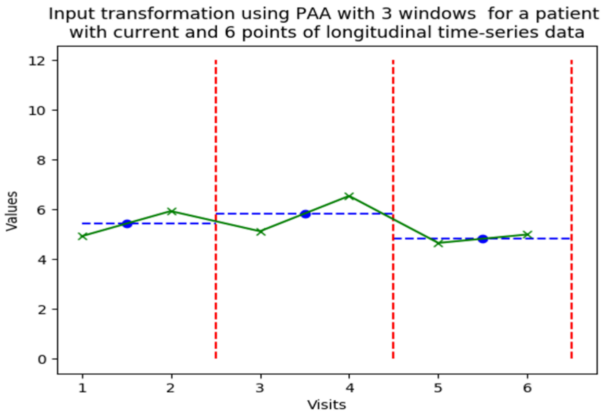


| Patient | Time step | $\tilde{x}_1$ |
|---|---|---|
| P2 | TS1 (current) | 4.72 |
| | TS2 | 5.44 |
| | TS3 | 5.84 |
| | TS4 | 4.83 |

Example of time series steps transformation using PAA for the cholesterol feature when the number of patient visits is more than $s = 3$. In this example, the number of visits is $r = 7$. The cholesterol value in the current visit is unchanged. The cholesterol values in the longitudinal time series data (6 visits) are transformed into 3 values using the PAA as shown in the bottom table.
