# Supplementary material for: Improving Current Glycated Hemoglobin Prediction in Adults: Use of Machine Learning Algorithms With Electronic Health Records
Source: JMIR Med Inform. 2021 May 24;9(5):e25237. doi: 10.2196/25237 (PMC8185616; doi:10.2196/25237)

Multimedia Appendix 5: AUC-ROC and PR-AUC curves for the models (with 10 folds) trained with longitudinal data.

Multiple Logistic Regression (MLR)

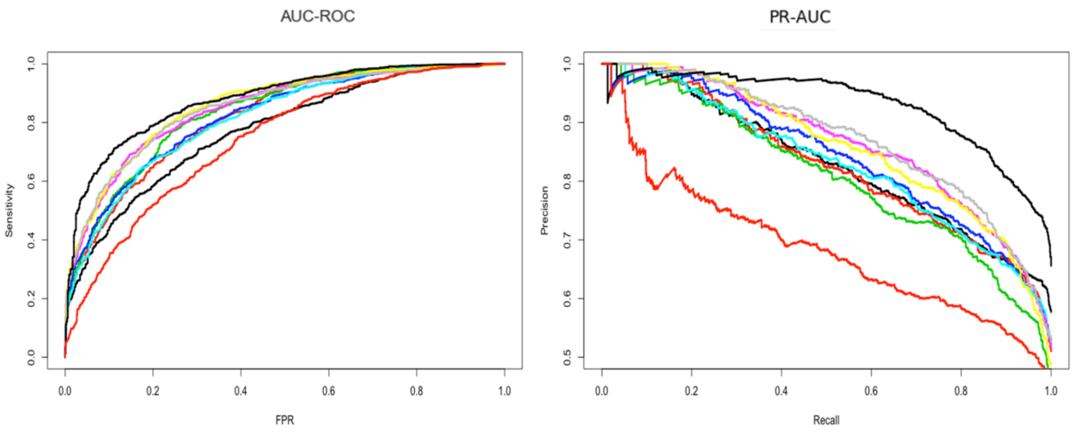

Random Forest (RF)

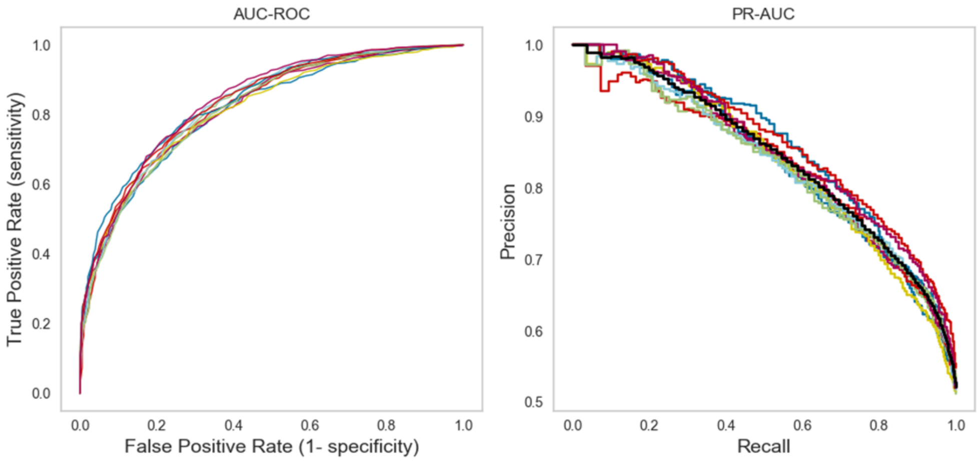

Logistic regression (LR)

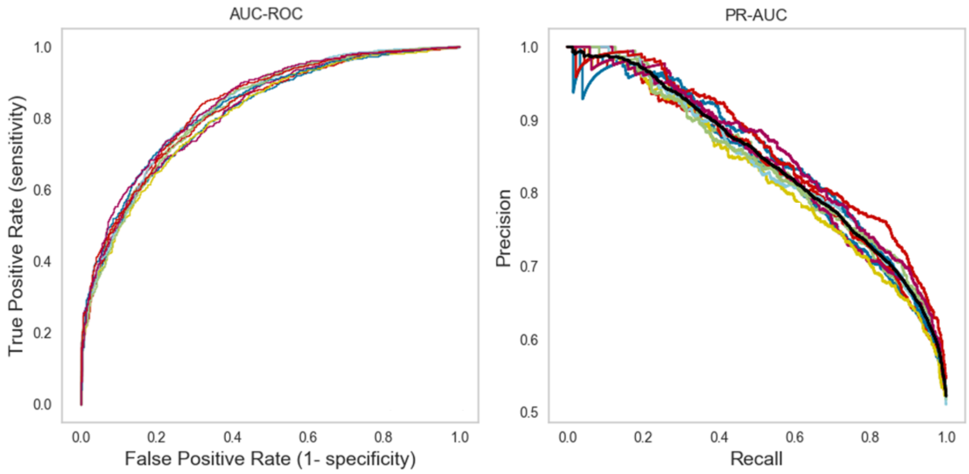

Support Vector Machine (SVM)

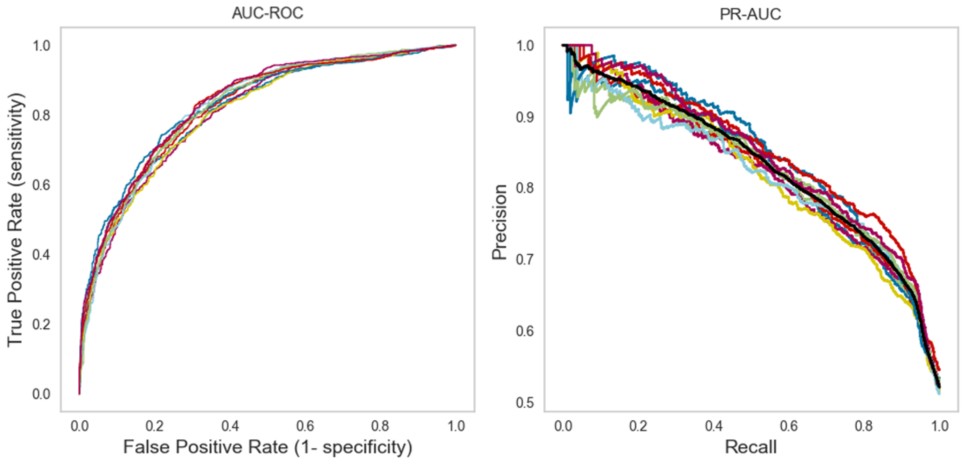

Multi-layer perceptron (MLP)

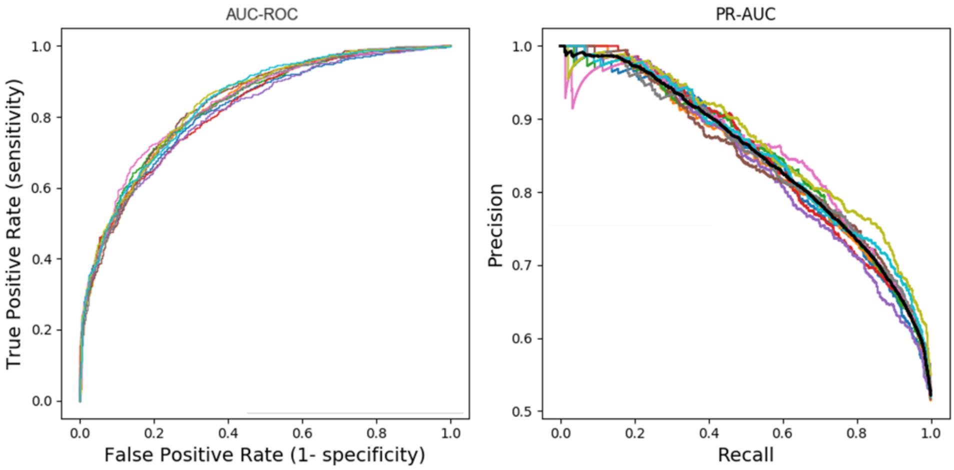

Supplement: Multimedia Appendix 5 [file medinform_v9i5e25237_app5.pdf]
