# Supplementary material for: Improving Current Glycated Hemoglobin Prediction in Adults: Use of Machine Learning Algorithms With Electronic Health Records
Source: JMIR Med Inform. 2021 May 24;9(5):e25237. doi: 10.2196/25237 (PMC8185616; doi:10.2196/25237)
Supplement: Multimedia Appendix 6 [file medinform_v9i5e25237_app6.pdf]

Multimedia Appendix 6

Variable relative importance charts for the models.

Multiple Logistic Regression (MLR)

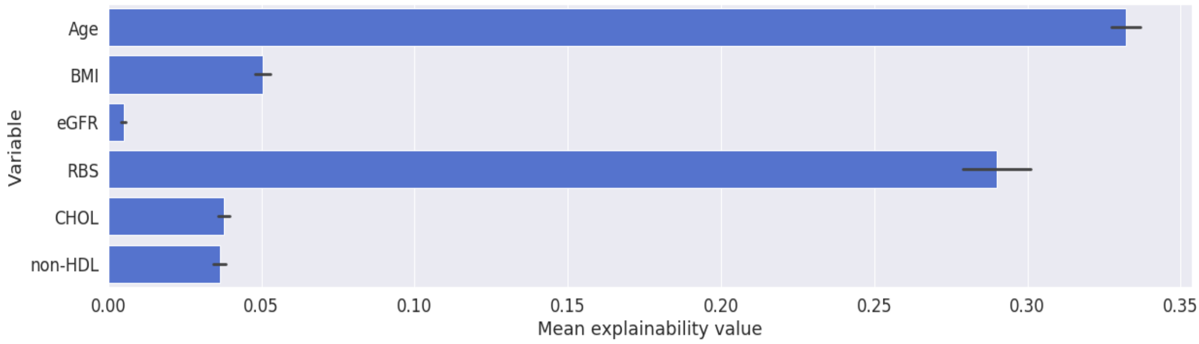

Order of importance of predictors for the MLR model trained without longitudinal data.

Random Forest (RF)

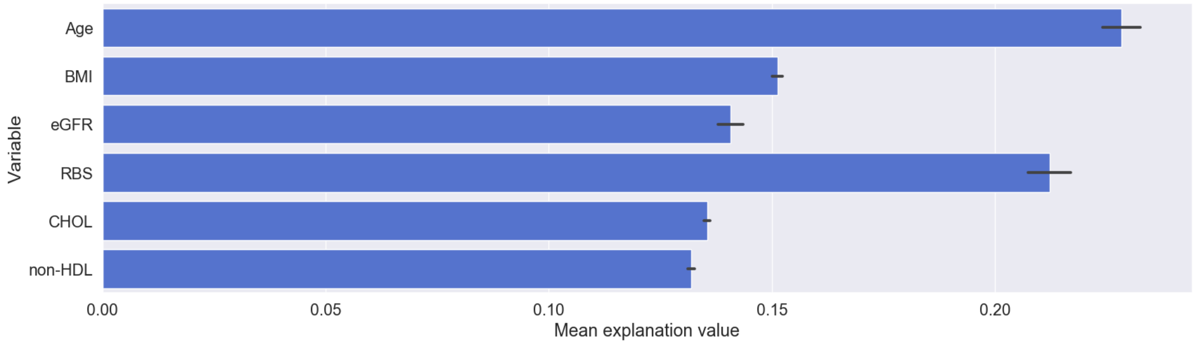

Order of importance of predictors for the RF model trained without longitudinal data.

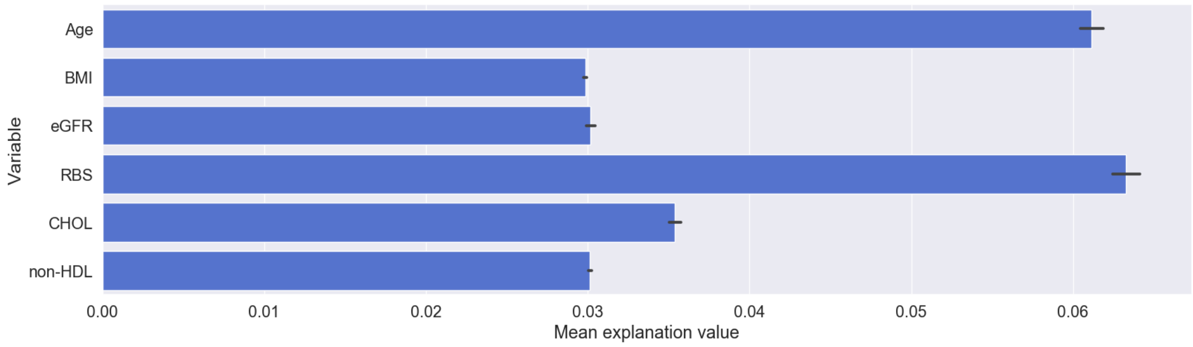

Order of importance of predictors for the RF model trained with longitudinal data.

Logistic regression (LR)

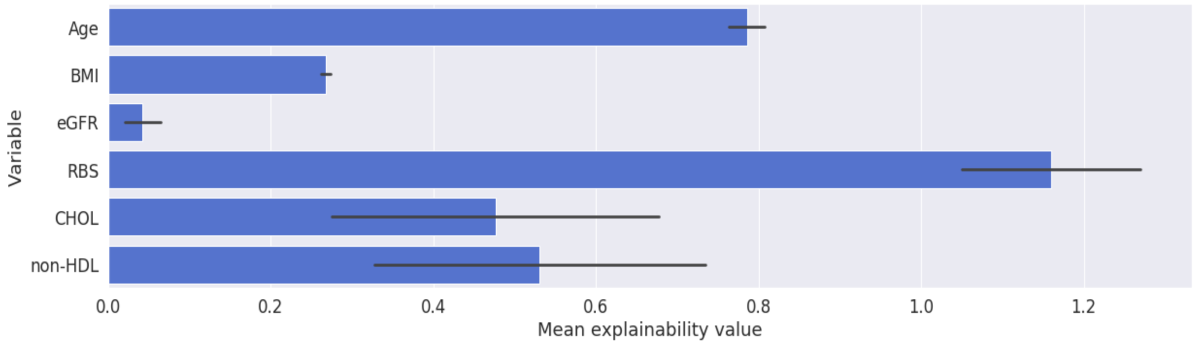

Order of importance of predictors for the LR model trained without longitudinal data.

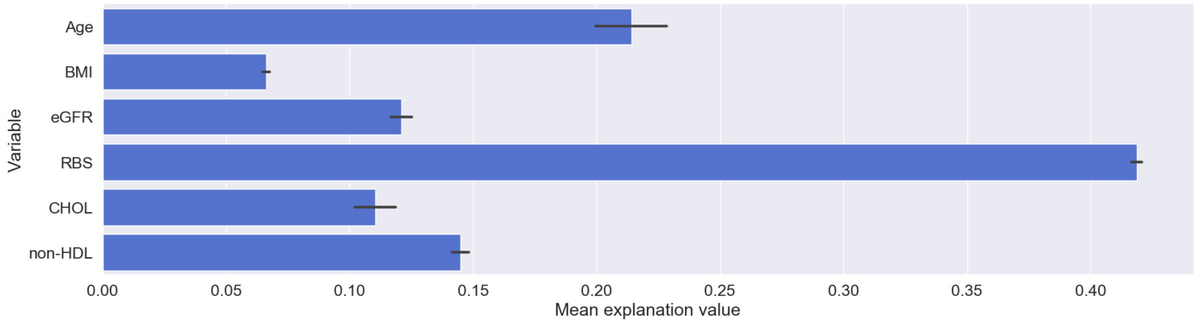

Order of importance of predictors for the LR model trained with longitudinal data.

Support Vector Machine (SVM)

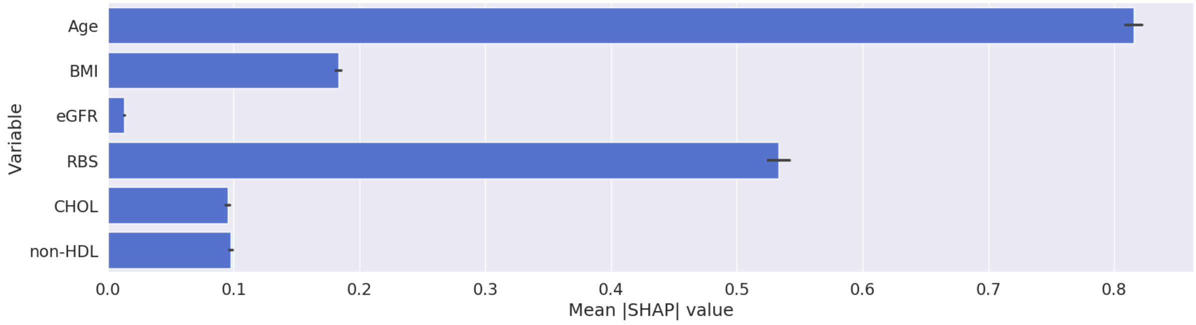

Order of importance of predictors for the SVM model trained without longitudinal data using SHAP.

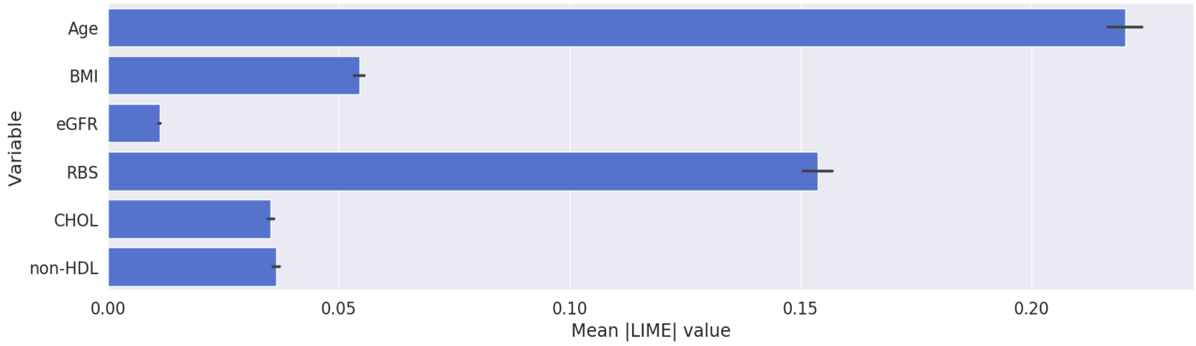

Order of importance of predictors for the SVM model trained without longitudinal data using LIME.

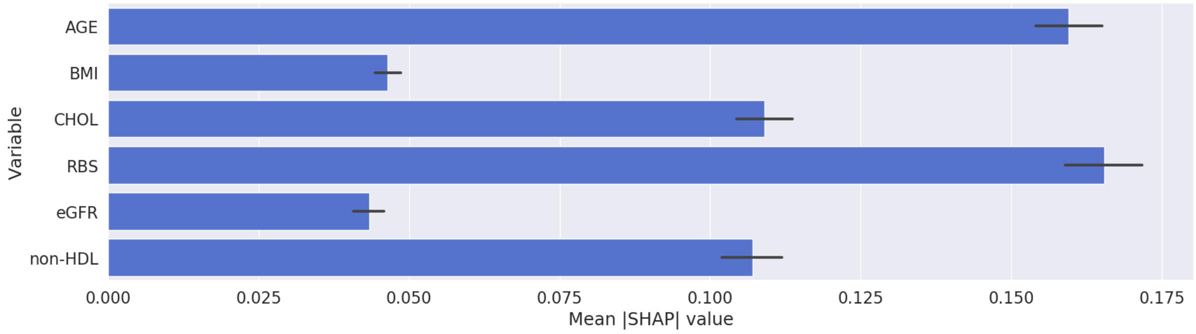

Order of importance of predictors for the SVM model trained with longitudinal data using SHAP.

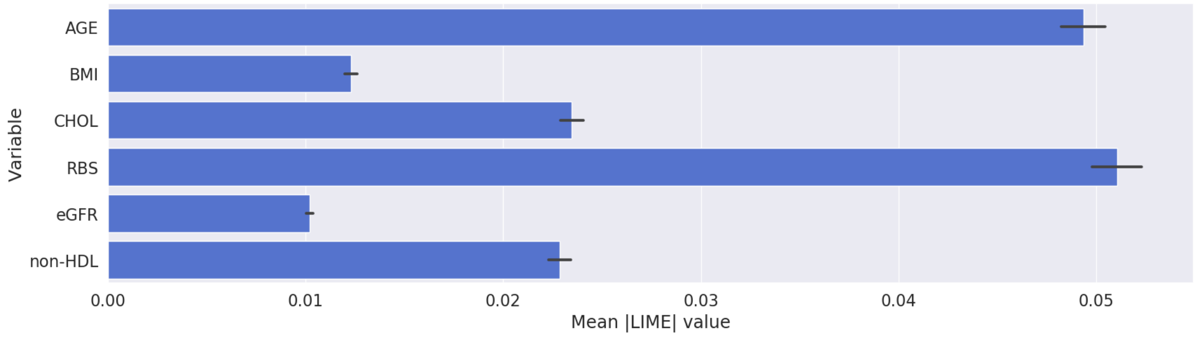

Order of importance of predictors for the SVM model trained with longitudinal data using LIME.

Multi-layer perceptron (MLP)

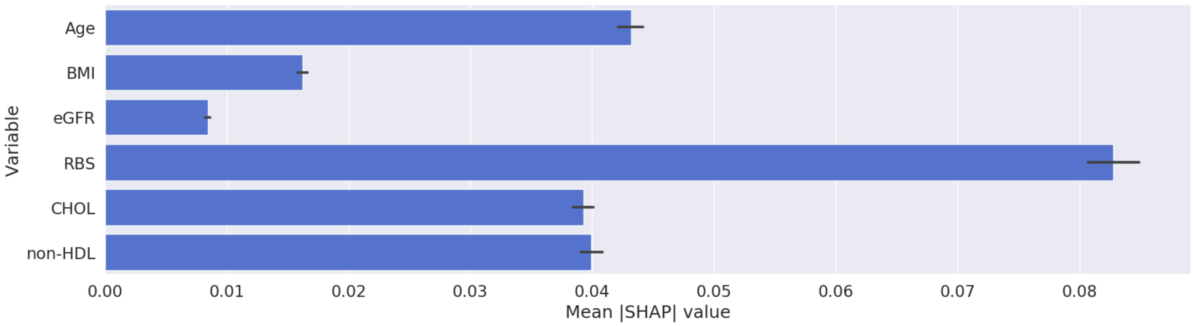

Order of importance of predictors for the MLP model trained without longitudinal data using SHAP.

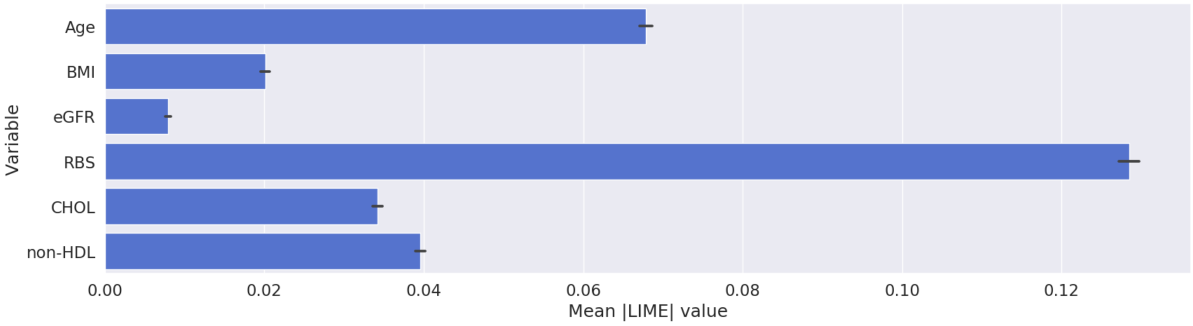

Order of importance of predictors for the MLP model trained without longitudinal data using LIME.

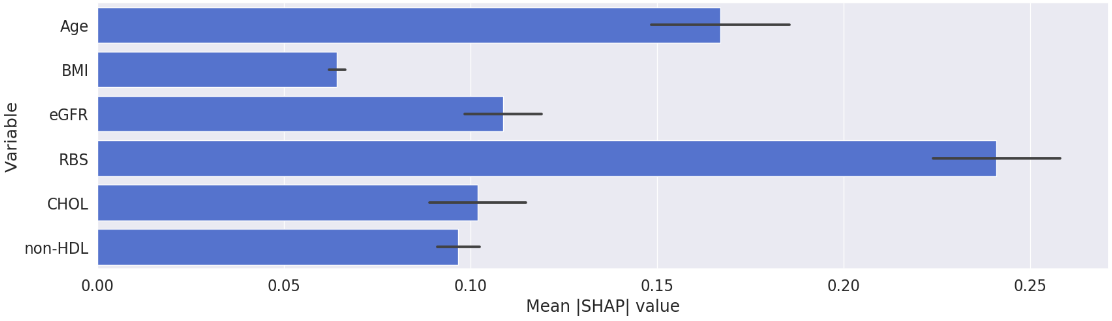

Order of importance of predictors for the MLP model trained with longitudinal data using SHAP.

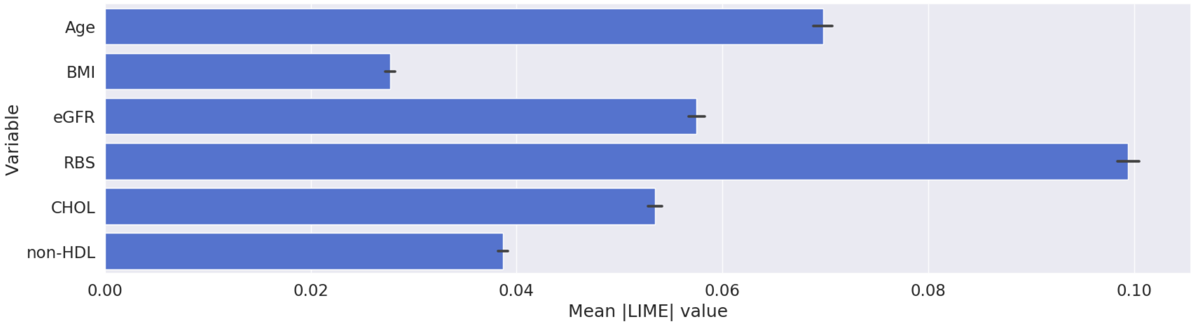

Order of importance of predictors for the MLP model trained with longitudinal data using LIME.
