# Supplementary material for: Improving Current Glycated Hemoglobin Prediction in Adults: Use of Machine Learning Algorithms With Electronic Health Records
Source: JMIR Med Inform. 2021 May 24;9(5):e25237. doi: 10.2196/25237 (PMC8185616; doi:10.2196/25237)
Supplement: Multimedia Appendix 7 [file medinform_v9i5e25237_app7.pdf]

Multimedia Appendix 7

Multiple Logistic Regression (MLR) model details

Table S1. The details of MLR trained without longitudinal data used for predicting HbA1c Elevation levels.

Intercept		
	-	7.4910884
Random Blood Sugar (Glucose) Level (RBS)		
	+	0.47325147* RBS
	-	0.0080969994 * max(RBS - 4.6,0)^3
	+	0.0099146931 * max(RBS - 5.7,0)^3
	-	0.0018176937 * max(RBS - 10.6,0)^3
estimated Glomerular Filtration Rate (eGFR)		
	+	0.0048805867 * eGFR
	-	1.1069511e-06 * max(eGFR - 51,0)^3
	+	2.8246337e-06 * max(eGFR - 96,0)^3
	-	1.7176827e-06 * max(eGFR - 125,0)^3
Body Mass Index (BMI)		
	+	0.055349506 * BMI
	-	8.3374022e-05 * max(BMI - 22.41,0)^3
	+	0.00014121475 * max(BMI - 29.07,0)^3
	-	5.7840728e-05 * max(BMI - 38.67,0)^3
Age		
	+	0.084259781 * AGE
	-	2.2417323e-05 * max(AGE - 29,0)^3
	+	4.3041261e-05 * max(AGE - 52,0)^3
	-	2.0623938e-05 * max(AGE - 77,0)^3
Total Cholesterol (CHOL)		
	-	0.77645878 * CHOL
	+	0.0086131708 * max(CHOL - 3.11,0)^3
	-	0.01640604 * max(CHOL - 4.44,0)^3
	+	0.0077928688 * max(CHOL - 5.91,0)^3
Non-High Density Lipoprotien (non-HDL)		
	+	0.71595282 * non_HDL
	+	0.0071327364 * max(non_HDL - 2.15,0)^3
	-	0.01264216 * max(non_HDL -3 .27,0)^3
	+	0.0055094239 * max(non_HDL - 4.72,0)^3

Logistic Regression (LR) details.

Table S2. The details of LR trained without longitudinal data used for predicting HbA1c Elevation levels.

Intercept		
	+	0.23815679
Random Blood Sugar (Glucose) Level (RBS)		
	+	1.06104915
estimated Glomerular Filtration Rate (eGFR)		
	-	0.0521671
Body Mass Index (BMI)		
	+	0.26236068
Age		
	+	0.75756277
Total Cholesterol (CHOL)		
	-	0.32541754
Non-High Density Lipoprotien (non-HDL)		
	+	0.36907342
